# Membrane Perfusion of Hydrophobic Substances Around Channels Embedded in the Contact Bubble Bilayer

**DOI:** 10.1038/s41598-017-07048-4

**Published:** 2017-07-31

**Authors:** Masayuki Iwamoto, Shigetoshi Oiki

**Affiliations:** Department of Molecular Physiology and Biophysics, University of Fukui Faculty of Medical Sciences, 23-3 Matsuokashimoaizuki, Eiheiji-cho, Yoshida-gun, 910-1193 Fukui Japan

## Abstract

In fluidic biomembranes, lipids and membrane proteins diffuse restlessly, and lipid compositions change steadily. To mimic dynamic behavior of the biomembranes, a method for introducing rapid changes in the constituents in the lipid bilayer was developed. In contact bubble bilayers (CBB), as a water-in-oil droplet bilayer system, the bilayer hydrophobic interior is contiguous with the bulk oil phase. Making use of this geometrical feature as an access route, hydrophobic substances were administered into the bilayer. Polytheonamide B, a cytotoxic hydrophobic peptide, was applied, and oriented incorporation and relevant single-channel current recordings were enabled. Nystatin was pre-loaded in the CBB, and sterol perfusion exhibited slow development of the macroscopic current. On the contrary, the reconstituted KcsA potassium channels immediately attenuate the channel activity when cholesterol was applied. This oil-phase route in the CBB allows rapid perfusion of hydrophobic substances around the bilayer-embedded channels during continuous recordings of channel currents.

## Introduction

Biological membranes are fluidic and mosaic^1–4^. Both phospholipids and membrane proteins diffuse in a two-dimensional space, where they interact intimately^5^. Lipids generate inhomogeneous phases^6^, while membrane proteins are often clustered with similar or different species in the membrane^7–9^. These dynamic and collective behaviors of lipid bilayer and membrane proteins underlie fundamental membrane functions in living cells^10, 11^. To understand these inter-relationships, experimental manipulations of lipid compositions are a prerequisite, but limited methods in biomembranes are available^12^. In lipid bilayer experiments^13–15^, even though lipid compositions can be arbitrarily controlled, they are not readily changed during experiments. Here, we developed a method for introducing rapid changes in the membrane constituents in the lipid bilayer experiments.

**Figure 1:**
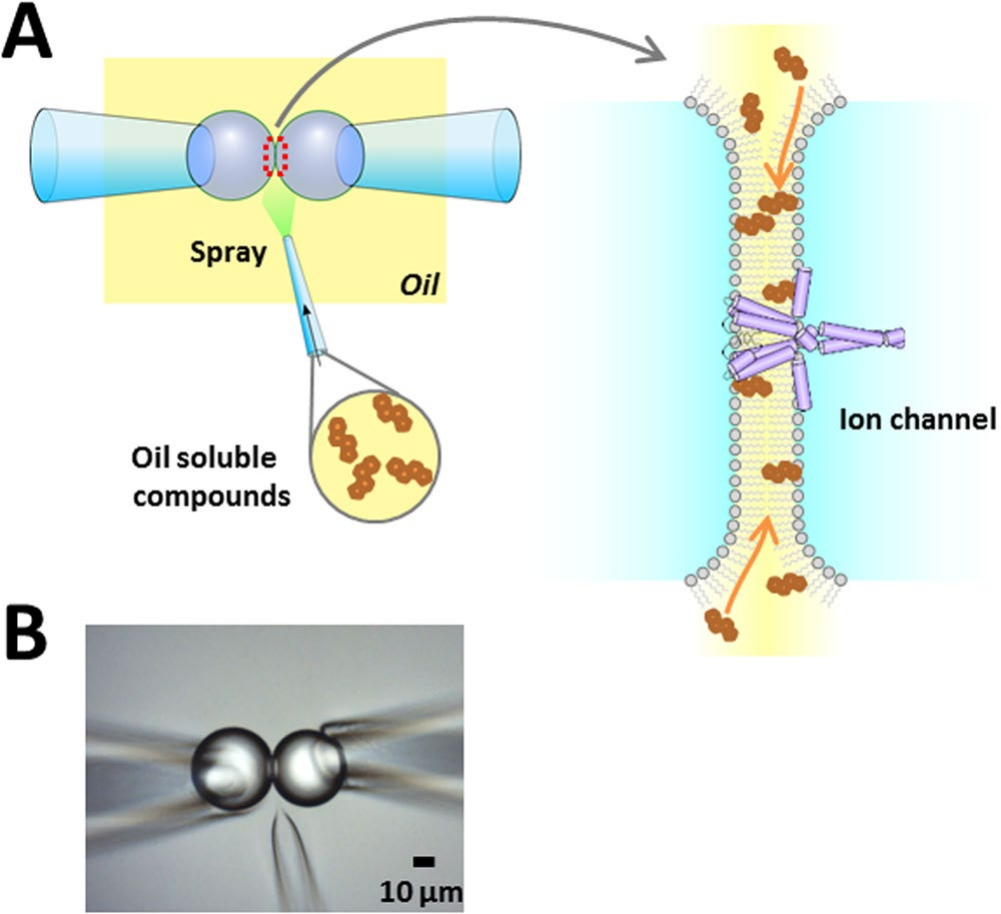
Membrane perfusion. (**A**) Scheme for membrane perfusion. In the CBB method, including other DIB methods, the bilayer interior is open to the bulk organic solvent phase. Thus, injection of hydrophobic substances in the oil phase transfers the substance to the bilayer membrane. During the perfusion, the channels as an integral membrane protein are anchored in the bilayer phase, and the hydrophobic substances perfuse around the membrane proteins. (**B**) A photo of the membrane perfusion system.

In the lipid bilayer methods, involving planar lipid^13, 16, 17^, droplet interface^18–22^, and contact bubble-bilayers^23^, the underlying physicochemical principle to form bilayers differ substantially from those of the *de novo* biomembrane formation. In contrast to the self-assembly of amphipathic phospholipids in the aqueous solution in the biomembrane, phospholipids, either dispersed in an organic solvent or reconstituted in liposomes, are used for forming phospholipid monolayers at the oil-water interface, and bilayers are formed from apposition of two monolayers^17, 24^. Once formed, the structures of biomembranes and lipid bilayers are indistinguishable, and extensive examination of channel function has exhibited equivalent activities in both membranes^25^. There is, however, one difference in the membrane organization between artificial lipid bilayers and biomembranes. As a consequence of the membrane formation procedure, the interior of the lipid bilayer is contiguous with the bulk organic solvent, meaning that the membrane interior is accessible from the bulk organic phase (Fig. 1). This is in contrast to the biomembrane, such that hydrophobic substances reach the membrane interior exclusively by partitioning from the aqueous solution.

In this study, we exploited this artificial oil-phase route in the CBB for introducing hydrophobic substances into the membrane interior. This route allows changes in the membrane composition in an unprecedented way. Hydrophobic substances having high oil solubility are injected into the oil phase close to the bilayer, which readily reach the lipid bilayer, by-passing the aqueous phase, via the hydrophobic route. In addition, we ensured that integral membrane proteins remain in the bilayer phase and do not transfer to the monolayer phase though they can freely move along the lipid bilayer. Accordingly, this method allows “perfusion” of hydrophobic substances around the membrane proteins embedded in the fluid membrane. Real time monitoring of channel activities upon perfusion of hydrophobic substances allows the experimental approach to elucidate dynamic channel-lipid interactions.

## Results

### The membrane perfusion method

The contact bubble bilayer (CBB; the oil phase was hexadecane; see Methods in detail)^23^ was used throughout this study, and phospholipids were mostly dissolved in the oil (hexadecane) phase. Easy manipulability of bubbles and the small membrane area (~10 *μ*m in diameter) were evidently beneficial for rapid perfusion. After formation of the CBB and incorporation of arbitrary channels, the bilayer membrane was voltage-clamped for steady channel recordings. By changing the channel concentration in the aqueous phase, the single-channel current or the macroscopic current can be recorded. A perfusion pipette having the tip diameter of a few *μ*m was filled with a hydrophobic substance which was dissolved in the same organic solvent (hexadecane) as that for the oil phase of the CBB system. The perfusion pipette was placed in the oil phase with its tip close to the bilayer ≤10 *μ*m; Fig. 1). By applying a small pressure (~0.2 kPa), the hydrophobic substance was squirt over the bilayer area with the flow rate of ~0.6 nL/s such that the bubbles were not blown off. Accordingly, the hydrophobic substance is partitioned into the monolayers and bilayer, and reaches the membrane embedded channels. This procedure allows rapid perfusion of the hydrophobic substance to the membrane^26^ via the oil-phase route, while recording channels currents for detecting immediate responses to the hydrophobic substance and their time courses. The perfusion can be continued, but once stopped the flow, the hydrophobic substance is partitioned back to the oil phase (see the section of “Effect of cholesterol on the KcsA potassium channel”).

### Access of pTB towards the membrane

**Figure 2:**
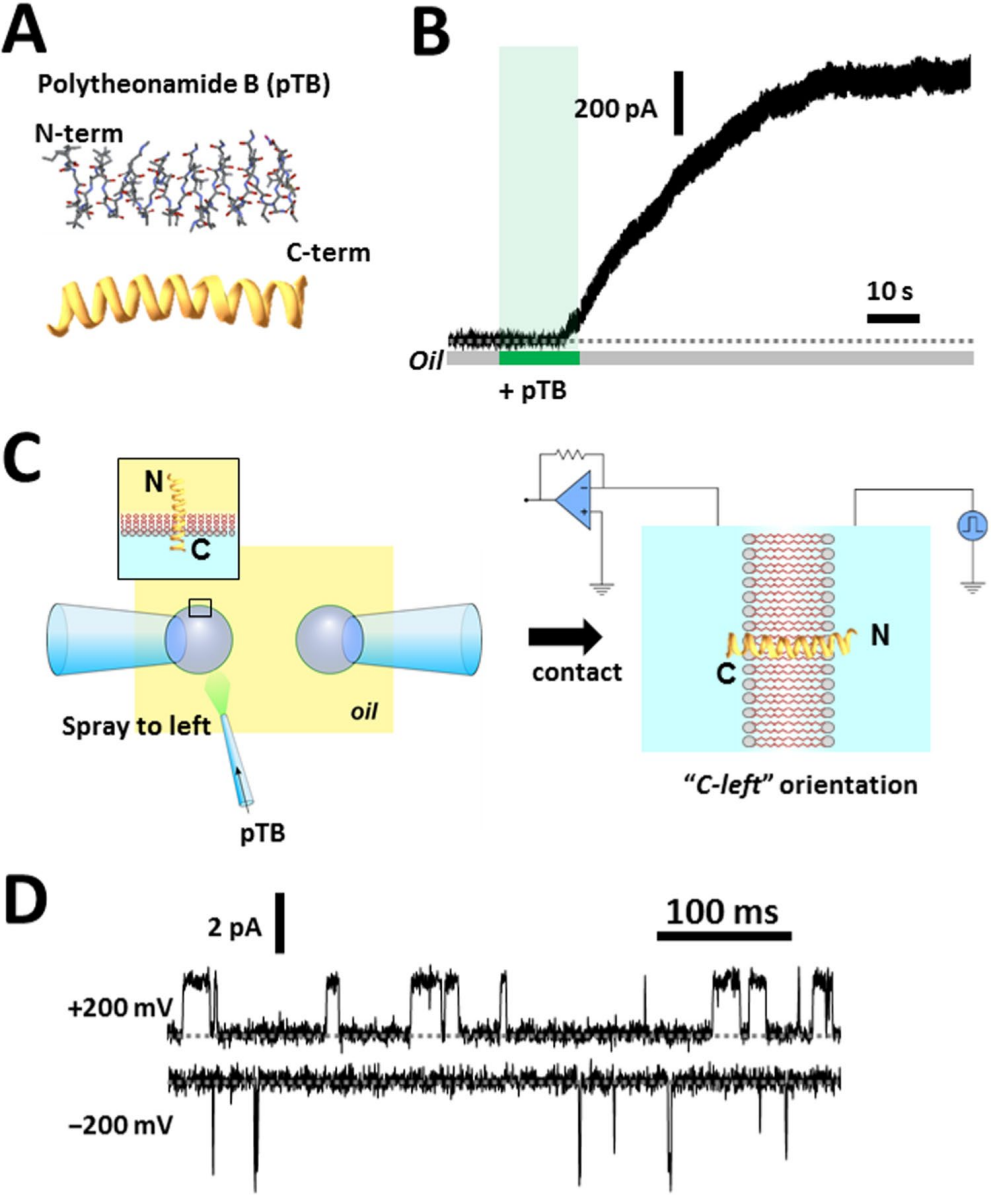
Channel molecule administration via the oil-phase route. (**A**) Structure of the pTB channel. (**B**) Administration of pTB at the vicinity of the DiphyPC membrane. pTB is a hydrophobic peptide, which was dissolved in hexadecane (50 nM) and applied to the oil phase. After application for 15 s, the channel current emerged after a delay and kept activating, even after stopping the perfusion, indicating that pTB partitioned into the monolayers was transferred to the bilayer. (**C**) Scheme for the vectorial insertion of pTB. When pTB was sprayed on one of the bubbles and the bubbles were attached, the orientation of the pTB channel in the membrane was controllable. Inset. The hydrophilic C-terminal rests in the aqueous phase, while the hydrophobic N-terminal stays in the hydrophobic phase. (**D**) The single channel current recording of the pTB channel. The channel current appeared immediately upon attaching the bubbles after spraying pTB (50 pM) to one of the bubbles. Conductance and open probability of the pTB channel is asymmetric to the applied membrane potential, consequently, the orientation is distinguishable.

First, to prove the presence of an effective oil-phase route to access the membrane interior, a peptide channel, polytheonamide B (pTB, Fig. 2A)^27, 28^, was used. pTB is a hydrophobic peptide (48 amino acid residues) extracted from the marine sponge, *Theonella swinhoei*. As a cytotoxic agent, pTB is released from the sponge into the aqueous environment and then targeted to the cell membranes. In this study, pTB was dissolved in hexadecane and applied to the oil phase in the vicinity of the bilayer (≤10 *μ*m; see Methods), which lead to spontaneous transfer into the bilayer via the hydrophobic route. The channel activities appeared after a substantial delay and macroscopic currents were recorded at high pTB concentration (50 nM; Fig. 2B). This is in contrast to the addition of pTB to the aqueous solution, in which the channel current is induced immediately^28^. The current steadily grew even after stopping the perfusion, which is an attribute of pTB having high affinity to the bilayer phase^28^. Either single-channel current or macroscopic currents were readily recorded, depending on the added pTB concentration.

To explore the mechanism of the membrane insertion, only one of the bubbles was exposed to pTB (one-bubble administration), followed by attachment of the two bubbles (Fig. 2C). The channel activity appeared immediately upon bilayer formation. Thus, the delayed activation, when added at the vicinity of the bilayer membrane, suggests that the bulky N-terminal moiety may hamper the transfer of pTB from the monolayer to the bilayer phase. When low concentration of pTB (50 pM) was applied with the one-bubble administration method, the active channels were inserted into the membrane in the same orientation (Table 1), which can be evaluated by the intrinsic asymmetric activity of the pTB channel^28, 29^ (Fig. 2D): Higher conductance was observed at negative voltages, and higher open probability was noted at positive voltages. These voltage dependent gating and the current rectification indicate that pTB is readily partitioned into the monolayer, and oriented with its hydrophilic C-terminal end resting in the aqueous phase. Once the pTB-doped monolayer docks with the pTB-free monolayer, the hydrophobic N-terminal end crosses the hydrophobic core of the membrane, and the membrane integrated pTB shows the channel activity with the defined orientation. This behavior is consistent with the phenomena that the orientation of the pTB channel is maintained even after the bilayer detachment followed by the attachment^23^.

**Table 1:** The orientation of the incorporated pTB channel after one-bubble perfusion.

Spray pTB to	C-left	C-right
Left bubble (n, 6)	100%	0%
Right bubble (n, 5)	0%	100%

### Cholesterol perfusion induced the nystatin current

**Figure 3:**
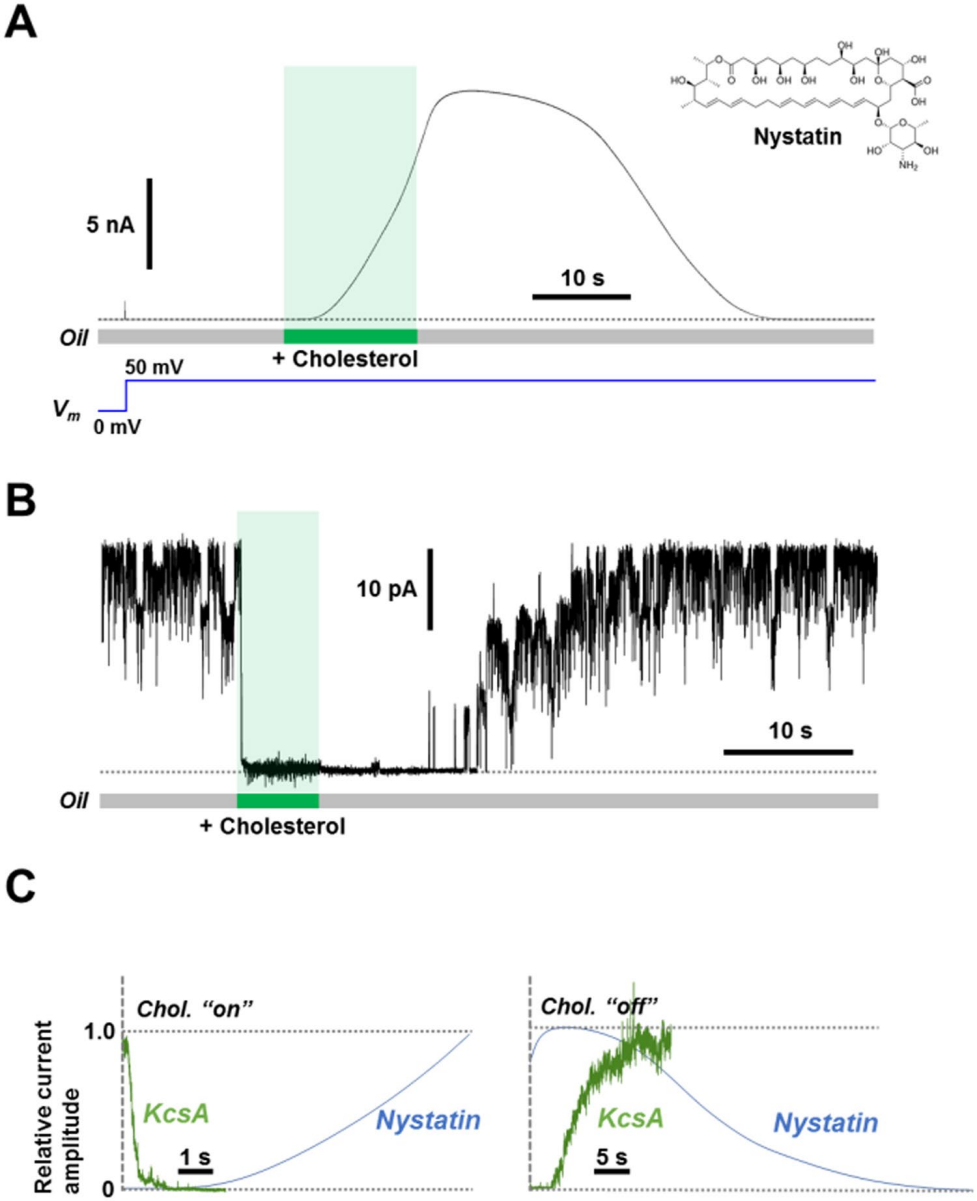
Membrane perfusion of cholesterol to nystatin- and KcsA channel-doped membranes. (**A**) Activation of the nystatin channel (50 *μ*g/mL) upon cholesterol (5 mg/mL) perfusion. Slowly increasing current implies multiple processes involved in the formation of multimeric channels with cholesterols. (**B**) Inhibitory effect of cholesterol on the KcsA potassium channel. Membrane contained several E71A mutant KcsA channels. The current was immediately attenuated upon cholesterol (5 mg/mL) perfusion. After stopping the perfusion, the channel current recovered to the original level, indicating that the number of the channels in the membrane was constant throughout the perfusion process. (**C**) The time courses of the channel activities of nystatin and KcsA to the cholesterol perfusion. The responses are shown to the cholesterol-on (left) and -off (right). The current amplitudes were normalized for the maximal currents.

To examine whether or not the oil-phase route is effective for changing a physiological membrane constituent, such as cholesterol, nystatin channel was used. Nystatin^30, 31^ is a polyene macrolide and a well-established ion channel that necessitates sterols in the membrane for the channel activity. The bilayer membrane was formed with the nystatin-doped liposomes added in the aqueous solution of a bubble (see Methods). No channel activity was measured at that time. The tip of the cholesterol-containing glass pipette (5 mg/mL) was taken close to the CBB, and cholesterol was ejected. The perfusion successfully induced the nystatin current (Fig. 3A). The current gradually increased, and even after stopping the perfusion of cholesterol, the current continued to increase. After a while, the current gradually decreased to zero level. The delayed activation and the delayed deactivation were reproducibly observed (n = 4). This indicates that cholesterol formed complexes with nystatin in the bilayer helping the assembly of active channels.

### Effect of cholesterol on the KcsA potassium channel

Various channel proteins are subjected to changes in their activities in the presence of cholesterol^32–34^. The mechanism of cholesterol actions still remains elusive for the KcsA potassium channel^35^. In addition, the time course of the channel-response to cholesterol has not been generally studied. The non-inactivating E71A mutant of KcsA channel^36^ was used for monitoring the response of the activation gate to cholesterol. The channels were reconstituted in the azolectin membrane, and Fig. 3B shows multiple channel activities. Upon perfusion of cholesterol, the KcsA channels immediately lost their activities, which is the first observation of such a fast response of a channel to cholesterol. In this experiment, cholesterol was perfused for 5 s. After stopping the perfusion, the channel activity gradually returned to the original level, indicating that the number of the membrane-embedded KcsA channels was constant before and after the cholesterol perfusion, since the channels are anchored within the bilayer phase. Also, the slow recovery indicates that the cholesterol concentration in the bilayer membrane decreased by partitioning back to the bulk oil phase.

### Time course of the cholesterol actions

The time course of the cholesterol action to nystatin and KcsA channels was compared (Fig. 3C). The immediate attenuation of the KcsA current upon cholesterol perfusion (Fig. 3B) indicates that transfer of cholesterol to the bilayer is rapid. Thus, the slow onset of the nystatin channel activity reflects the time course for nystatin-cholesterol encounter and binding to form an assembled channel structure. An extremely slow time course of attenuation after removal of cholesterol suggests that the nystatin-cholesterol binding is tight. In contrast, the inhibitory action of cholesterol on the KcsA channel was immediate, and the recovery was also relatively fast, suggesting that cholesterol does not bind tightly to the KcsA channel. The mechanism underlying these kinetic differences will be examined in future studies. In contrast to hydrophobic substances, perfusion of a negatively charged phospholipid, phosphatidylglycerol (PG), via the oil-phase route was examined, which is shown in the Supporting Information (Fig. S1).

## Discussion

**Figure 4:**
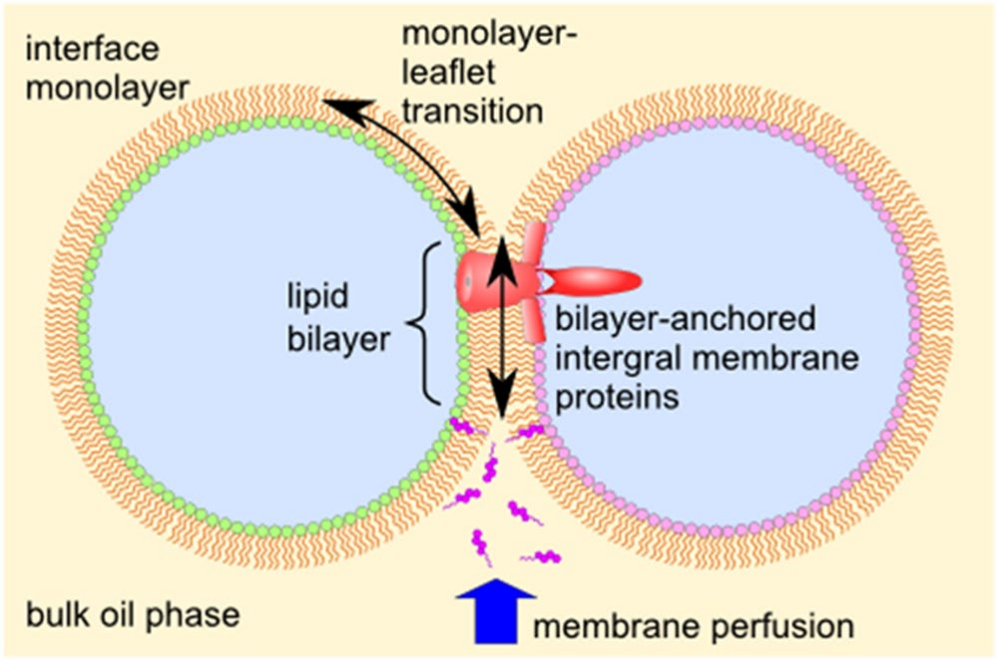
A schematic representation of the water-in-oil droplet system, including the CBB. The lipids in the interface monolayer and those in a leaflet of the bilayer are freely diffusible (monolayer-leaflet transition). Integral membrane proteins, such as channels, freely diffuse but within the bilayer (bilayer-anchored). Hydrophobic substances perfuse around the membrane embedded channels via partitioning to either monolayer or bilayer.

In this study, we established the method of membrane perfusion via the oil-phase route. Hydrophobic substances are incorporated into the bilayer either through direct access to the bilayer phase or partitioning into monolayers and then transferring to the bilayer phase. This oil-phase route is a bypass for the hydrophobic substance, and opens the membrane interior to the external world. Highly hydrophobic substances with poor solubility in aqueous solutions, involving pharmacological substances, can be readily delivered to the membrane interior using this method. In the CBB method involving other droplet interface bilayers (DIB), the following shared features rendered the membrane perfusion readily usable (Fig. 4):

- Each leaflet of the membrane is an open system, contiguous with the bulk monolayer of each bubble.
- The organic solvent phase is in contact with the membrane interior.
- Integral membrane proteins are ‘anchored’ in the bilayer phase.
- One-bubble administration is effective, as long as the frequency of flip-flop of the relevant substance is low.

In addition, small bubble size (~50 *μ*m), and consequently small bilayer size, of the CBB allows spraying of substances covering the entire bilayer circumference, leading to rapid perfusion of the membrane composition. Contrarily in the case of the DIB, a spray from the perfusion pipette does not cover the entire circumference of the large bilayer, and the “perfusion” was inefficient.

Manipulating bilayer membranes in each leaflet level has been developed, which advanced membrane science substantially. Asymmetric membranes having distinct lipid composition can be readily formed by the ‘interface passing’ method^37–39^, the DIB^40–42^ and CBB. However, mechanical manipulation of detach-attach of leaflets is readily controlled in the CBB^23^. Consequently, in the hydrophobic perfusion method, hydrophobic substances can be added in one of the leaflets (one-bubble exposure). Manipulating bilayer membranes at the leaflet level constitutes a monolayer technology, which opens more analytical and constitutive ways for study of the membrane.

## Methods

### Reagents

All chemicals, except for the phospholipids and Nystatin, were purchased from Nacalai Tesque (Kyoto, Japan). Azolectin (L-*α*-phosphatidylcholine type IV-S) was obtained from Sigma–Aldrich (St. Louis, MO, USA), and the other phospholipids (POPC, POPG, and DiphyPC) were obtained from Avanti Polar Lipids (Alabaster, AL, USA). Nystatin was purchased from Santa Cruz Biotechnology, Inc. (Dallas, TX, USA).

### Sample preparation

Purified polytheonamide B (pTB) was a kind gift from Dr. S. Matsunaga (Univ. Tokyo, Japan). The expression, purification, and reconstitution into liposomes of the KcsA channels has been described elsewhere^43^. Proteoliposomes were prepared by dilution as follows. First, liposomes prepared using azolectin were suspended in 200 mM KCl at a concentration of 2 mg/mL. Then, an aliquot of solubilized KcsA channel in 0.06% n-dodecyl-*β*-D-maltoside (DDM) was diluted 50 times with the liposome solution. The lipid/protein weight ratio of the proteoliposome was 2000. The proteoliposome suspension was mixed with a small amount of concentrated buffer (pH 7.5 or 4.0) just before the experiments.

### Contact bubble bilayer (CBB) experiments

The CBB method has been described in detail elsewhere^23^. Glass pipettes for the bubble formation and perfusion were fabricated from borosilicate capillary glass (OD/ID; 1.50/1.05 mm, Hilgenberg GmbH, Malsfeld, Germany) using a micropipette puller (P-87, Sutter Instrument, Novato, CA, USA). For the bubble-forming pipette, the tip of the pipette was broken and slightly polished using a micro-forge (MF-830, Narishige, Tokyo, Japan), obtaining a tip diameter of 30 *μ*m. All experiments were performed on an inverted microscope (IX73, Olympus, Tokyo, Japan). The pipettes were operated by motor-driven micromanipulators (EMM-3NV, Narishige) under the microscope. The pressure in the bubble-forming pipettes was regulated by a pneumatic microinjector (IM-11-2, Narishige) and the perfusion was regulated by a micro-volume perfusion system (*μ* Flow, ALA Scientific, Farmingdale, NY, USA).

In a concaved slide glass set on the microscope stage, a small amount of hexadecane was filled. The liposome or proteoliposome solution (2 mg/mL) of desired lipid composition was filled in the bubble-forming pipette, and the tip was soaked in hexadecane. The liposome solution was blown at the tip of the pipette by applying pressure inside the pipette to form small water-in-oil bubble coated with lipid monolayer. Two bubbles contacted each other by pipette manipulation, forming the contact bubble bilayer (CBB). Ag/AgCl wire electrodes (E255, Warner Instruments, Hamden, CT, USA) were set inside the bubble-forming pipettes, and the ionic current across the CBB was measured under the membrane voltage-clamped condition using a patch clamp amplifier (EPC800USB, HEKA, Lambrecht/Pfalz, Germany). The current signals were filtered (1 kHz for the cutoff frequency) and sampled at 5 kHz by an A/D converter (Digidata 1550 A, Molecular Devices, Sunnyvale, CA, USA) and stored in PC using a software (pCLAMP, Molecular Devices).

In the pTB experiments, CBB of DiphyPC was prepared in advance. Aliquots of pTB solution (1 nM or 1 *μ*M in ethanol) were suspended in hexadecane (final concentration, 50 pM or 50 nM) and used for the perfusion. In the nystatin experiments, 50 *μ*g/mL of nystatin was added beforehand to the liposome solution of POPC. In the KcsA experiments, pH of the proteoliposome solutions were set asymmetric to 7.5 and 4.0, and the reference electrode was connected to the pipette of pH 7.5 side^43^. Cholesterol was dissolved in hexadecane to a final concentration of 5 mg/mL and used for perfusion.

## Electronic supplementary material

Supplementary Information
