# Supplementary material for: Membrane Perfusion of Hydrophobic Substances Around Channels Embedded in the Contact Bubble Bilayer
Source: Sci Rep. 2017 Jul 31;7:6857. doi: 10.1038/s41598-017-07048-4 (PMC5537337; doi:10.1038/s41598-017-07048-4)
Supplement: Supplementary file 1 — Supplementary Information [file 41598_2017_7048_MOESM1_ESM.pdf]

Supplementary Information

Membrane Perfusion of Hydrophobic Substances Around Channels Embedded in the Contact Bubble Bilayer

*Masayuki Iwamoto & Shigetoshi Oiki**

[1]Department of Molecular Physiology and Biophysics, University of Fukui, 23-3 Matsuokashimoaizuki, Yoshida-gun, Fukui 910-1193, Japan.

Effect of PG on the KcsA potassium channel embedded in the PG-free membrane

As a control experiment, the KcsA potassium channel was examined for the effect of PG, since it has been thoroughly examined. PG was added either in the oil phase for the membrane perfusion or in the aqueous bubble as PG liposomes. The bilayer membrane was formed with phosphatidylcholine (PC), in which the KcsA channel shows very low open probability. Thus, incorporation of PG in the bilayer activates the channel gating in whatever route is taken to access to the bilayer. When PG was dissolved in hexadecane and flushed into the KcsA-reconstituted membrane, the current gradually increased. In contrast, when the PG-containing liposomes were perfused in the aqueous solution, there was rapid increase in the current. Thus, for charged lipids, the aqueous route is more appropriate to access the bilayer.

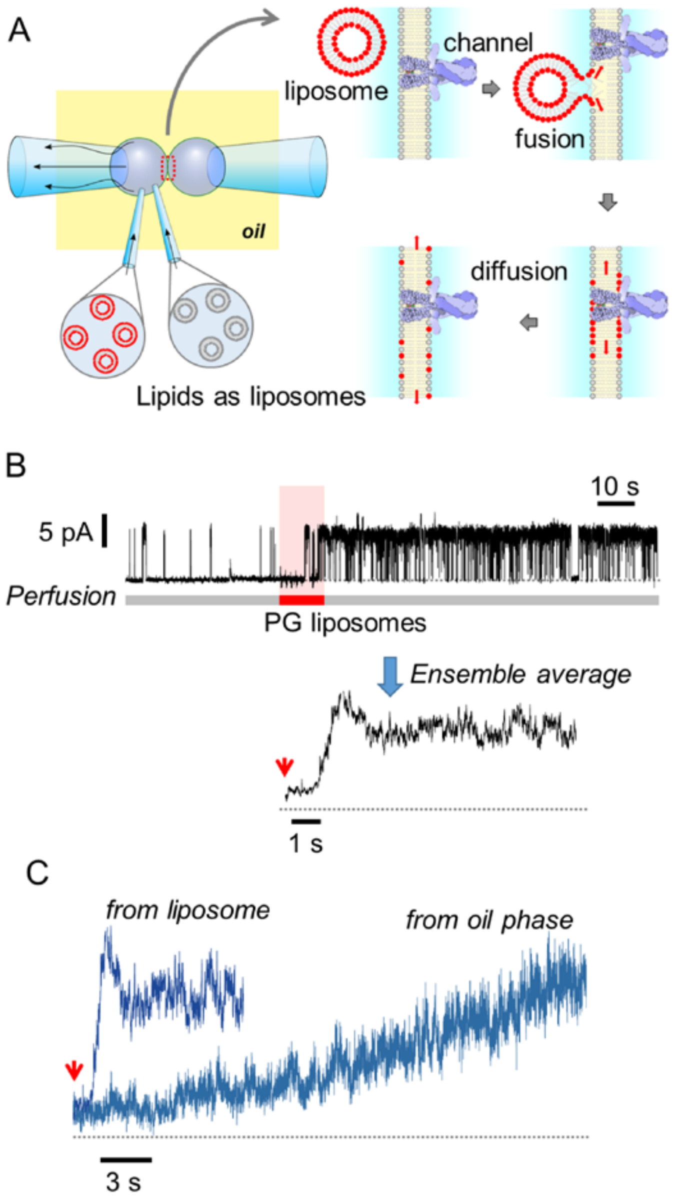

Fig. S1. Effect of PG on the KcsA channel. (A) A schematic representation of PG administration via liposome fusion. Liposomes were formed with PG, which were injected into one of the bubbles. (B) Experimental results of the PG liposome on the KcsA channel embedded in PC membrane. The E71A mutant of the KcsA channel showed low open probability in the single-channel current recordings before the PG administration. Immediately after the addition of the PG liposome, the channel increased the open probability. The single-channel currents were ensemble averaged to show the time course of the activation. (C) Membrane perfusion of PG and the activation of the KcsA channel. In contrast to the PG liposome, membrane perfusion of PG gradually increased the channel current. Experiments were performed with the E71A mutant of the KcsA channel in 200 mM KCl (pH 7.5 for the extracellular side and pH 4.0 for the intracellular side.) at +100 mV.
